# Protonation-Induced Enhanced Optical-Light Photochromic Properties of an Inorganic-Organic Phosphomolybdic Acid/Polyaniline Hybrid Thin Film

**DOI:** 10.3390/nano10091839

**Published:** 2020-09-15

**Authors:** Qingrui Zeng, Suyue Guo, Yuanbo Sun, Zhuojuan Li, Wei Feng

**Affiliations:** 1 Key Laboratory of Groundwater Resources and Environment, Ministry of Education, College of New Energy and Environment, Jilin University, Changchun 130021, China; Zengqr36776@163.com (Q.Z.); Guosy36776@163.com (S.G.); lizhuojuan36776@163.com (Z.L.); 2 College of Earth Science, Jilin University, Changchun 130061, China; Sunyb36776@163.com

**Keywords:** phosphomolybdic acid (PMoA), polyaniline (PANI), protonation, photochromism, nanocomposite thin film

## Abstract

A phosphomolybdic acid/polyaniline (PMoA/PANI) optical-light photochromic inorganic/organic hybrid thin film was successfully synthesized by protonation between the the multiprotonic acid phosphomolybdic acid (H_3_PO_4_·12MoO_3_) and the conductive polymer polyaniline. The stable Keggin-type structure of PMoA was maintained throughout the process. Protonation and proton transfer successfully transformed the quinone structure of eigenstate PANI into the benzene structure of single-polarized PANI in the PMoA/PANI hybridized thin film, and proton transfer transformed the benzene structure of single-polarized PANI back to the quinone structure of eigenstate PANI in the PMoA/PANI hybrid thin film, as verified by Fourier transform infrared spectroscopy (FTIR) and X-ray photoelectron spectroscopy (XPS). The average distribution of PMoA/PANI was observed by atom force microscopy (AFM). Interestingly, protonation of PMoA caused PANI to trigger transformation of the quinone structure into the single-polarized benzene structure, which enhanced the electron delocalization ability and vastly enhanced the maximum light absorption of the PMoA/PANI hybrid thin film as confirmed by density functional theory (DFT), electrochemistry, and ultraviolet-visible spectroscopy (UV-Vis) studies. Under optical-light illumination, the pale-yellow PMoA/PANI hybrid thin film gradually turned deep blue, thus demonstrating a photochromic response, and reversible photochromism was also observed in the presence of hydrogen peroxide (H_2_O_2_) or oxygen (O_2_). After 40 min of optical-light illumination, 36% of the Mo^5+^ species in PMoA was photoreduced via a protonation-induced proton transfer mechanism, and this proton transfer resulted in a structural change of PANI, as observed by XPS, generating a dominant structure with high maximum light absorption of 3.46, when compared with the literature reports.

## 1. Introduction

Photochromism is a unique physical-chemical phenomenon that is widely applied in various fields, such as information presentation [1], photodriven nanomachines [2], optical switching [3], optical memory devices and sensors [4,5,6], high-density optical-electron information storage [7], and molecular recognition or examination [8]. Since single-system photochromic materials exhibit poor photochromic properties, such as poor reversibility, low photochromic activity after coloring, narrow spectral response, fatigability, and color monotony, photochromic materials that can easily overcome these limitations have attracted scientists’ attention. Koski et al. [9] synthesized a kind of inorganic/inorganic photochromic material by intercalating the zero-valent metals Sn and Co into MoO_3_ nanoribbons, resulting in a change in the of color MoO_3_ from transparent white to dark blue and demonstrating the potential of this system in intelligent color windows and convenient color change sensors. Li et al. [10] fabricated a semiconductor/semiconductor-type MoO_3_@TiO_2_ crystalline-core amorphous-shell nanorod photochromic material and the unique structure and heterojunction formed between MoO_3_ and TiO_2_ enhanced the photochromic properties of MoO_3_. Dai et al. [11] synthesized a doped inorganic-type photochromic material by doping Pb^2+^ into LiEuMo_2_O_8_, which expanded and distorted the crystal cell of LiEuMo_2_O_8_, resulting in a 50% enhancement in the red light emission from LiEuMo_2_O_8_. Lu et al. [12] synthesized a new type of inorganic/organic hybrid photochromic material, As_4_Mo_8_O_33_/C_3_N_2_H_5_, which showed a remarkable photochromic effect, indicating that proton transfer from the imidazole cation to the polyanion plays a key role in the photochromic process.

Polyoxometalates (POMs) are widely applied photochromic chemicals with complex morphological characteristics and unique physical-chemical properties, leading researchers to focus on developing many types of photochromic composites [13]. Posphomolybdic acid (H_3_PMo_12_O_40_, PMoA), a representative Keggin-type polyoxometalate and polyprotic acid, possesses many advantages characteristic of photochromic materials, such as a large molecular volume, high thermal stability, maintenance of the crystal structure regardless of whether the material exists in the solid or liquid state, strong electron and proton storage capacities, and biocompatibility with organisms and it forms “organic-polyacidic compound anions”-type electron donor and electron acceptor hybrid materials. Feng et al. synthesized a series of organic-polyacidic compound anion-type photochromic materials, such as PMoA/polyacrylamide (PAM), PMoA/polyvinyl pyrrolidone (PVP) [14], PMoA/polyvinyl alcohol resin (PVA) [15], and PMoA/polyethylene glycol (PEG) [16], which can easily combine with PMoA to form “organic-polyacidic compound anion” hybrid films for diverse uses. However, the poor photochromic properties of these materials because of their functional groups and poor electron conversion properties limit their application. In addition, Xiao et al. [17] fabricated the Ni/Na inorganic/organic hybrid supermolecule NiEDTA-PW_12_, which exhibited rapid and reversible photochromism and strong antifatigue properties. Li et al. [18] fabricated polyvinyl pyrrolidone (ppy)/hexatungstate (HTA) and applied it to filter paper, realizing rapid and reversible color conversion.

Polyaniline (PANI) is a kind of industrial conductive polymer with unique electrical properties [19], optical properties [20], and magnetic properties [21,22]. Furthermore, as a kind of conductive polymer, PANI also easily undergoes protonation in the presence of a protonic acid, which will change the electrical conductivity and improve its photochromic properties, thus increasing its application value [19,20,23]. In this work, PMoA was combined with PANI to form an inorganic-organic hybrid photochromic PMoA/PANI hybrid film. PMoA, a polyprotic acid, could easily have a protonation effect on PANI, which would significantly change the conductivity of PANI. We explored the protonation effect of PMoA on PANI and the physical-chemical change of the PMoA/PANI hybrid film during the photochromic process through atomic force microscopy (AFM), Fourier transform infrared spectroscopy (FTIR), X-ray photoelectron spectroscopy (XPS), electrochemical studies, ultraviolet-visible (UV-vis) spectroscopy and density functional theory (DFT) calculations, and these techniques were also used to elucidate the photochromic mechanism of the PMoA/PANI hybrid film. This characterization demonstrated that the protonation effect of PMoA on PANI played a significant role in enhancing proton conversion and the high maximum visible light absorbance of this photochromic material.

## 2. Experimental

### 2.1. Substrate Modification

We used ***piranha*** solution, which is a kind of superoxidant, to modify the hydrophilic substrates. The ***piranha*** solution consisted of a mixture of concentrated sulfuric acid (98%, analytical reagent, Sinopharm Chemical Reagent Co. Ltd., Shanghai, China) and hydrogen peroxide (30%, analytical reagent, Shanghai Macklin Reagent Co. Ltd., Shanghai, China) with a volume ratio of 7:3. Quartz substrates (20.0 mm × 15.0 mm × 1.00 mm) were treated with ***piranha*** solution at 353 K for 24 h and then rinsed repeatedly with deionized water followed by ethanol (analytical reagent, Shanghai Macklin Reagent Co. Ltd., Shanghai, China); after that, the substrate was stored in acetone (analytical reagent, Shanghai Macklin Reagent Co. Ltd., Shanghai, China). After the acetone is completely volatilized, the quartz substrate can be used. A silicon wafer (20.0 mm × 15.0 mm × 1.00 mm) was treated with the same procedure for AFM and XPS characterization. A KBr pellet (radius of 10.0 mm, thickness of 2.00 mm) was formed from KBr powder (analytical reagent, Shanghai Macklin Reagent Co. Ltd., Shanghai, China) by using a tablet press and was used for the FTIR experiments.

### 2.2. Preparation of the PMoA/PANI Hybrid Thin Film

**Figure 1:**
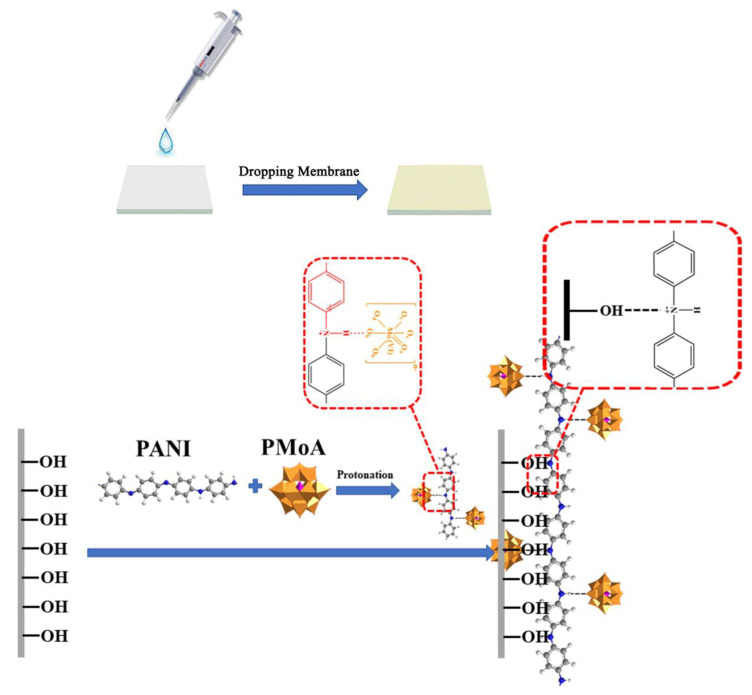
The protonation process to fabricate a PMoA/PANI hybridizing thin film.

The double crystal H_3_PMo_12_O_40_ (PMoA, analytical reagent, Shanghai Reagents Co. Ltd., Shanghai, China) was stored until use. A 0.02 g aliquot of polyaniline (PANI 98%, Shanghai Macklin Biochemical Co. Ltd., Shanghai, China) was mixed with 20 mL of a mixture of *N*,*N*-dimethylformamide (DMF, analytical reagent, Shanghai Macklin Reagent Co. Ltd., Shanghai, China) and ethanol to form solution No. 1 with a density of 0.1 mg/mL. A 0.04 g aliquot of H_3_PMo_12_O_40_ (PMoA, analytical reagent, Shanghai Reagents Co. Ltd., Shanghai, China) was mixed with 20 mL of a mixture of *N*,*N*-dimethylformamide (DMF, analytical reagent, Shanghai Reagents Co. Ltd.) and ethanol (analytical reagent, Shanghai Reagents Co. Ltd.) to form solution No. 2 with a density of 0.2 mg/mL. Solution No. 1 and solution No. 2 were mixed to form solution No. 3. A pipetting gun was used to drop this mixed solution onto a quartz substrate to form a PMoA/PANI hybrid thin film with a amount of 100 μL, as shown in Figure 1. To test the thin film thickness, solution No. 3 was dropped on a quartz substrate to form a thin film, than the quartz substrate was put on the test bed of an thin film thickness measurement system (FCT-1030, Changchun Institute of Optics, Fine Mechanics and Physics, LCD Lab, Chinese Academy of Science. Changchun, China). When visible light is vertically illuminated on the measured film, one part of the light is reflected on the surface of the film, the other part penetrates into the film and then reflects at the interface between the film and the bottom layer. The thickness of the hybrid thin films was nearly 1.8 μm, as calculated by the supporting software of the thin film thickness measurement system.

### 2.3. Characterization

AFM experiments were performed on a 300HV atomic force microscope (Seiko, Tokyo, Japan). FTIR spectroscopy was performed on a 550 Fourier transform infrared spectrometer (Nicolet, Madison, WI, USA) over a wavenumber range of 500–4000 cm^−1^. XPS analyses were performed on an ESCALAB 250 spectrometer (Thermo, Waltham, MA, USA) with an AlKα (1486.6 eV) and MgKα (1253.6 eV) mono X-ray source. Photochromic property measurements were performed on a V-500 UV-optical spectrophotometer (JASCO, Tokyo, Japan) in the range of 350–900 nm with an optical detection resolution of 1 nm. Electrochemical experiments were performed on a CHI660E electrochemical workstation (Chinstruments, Shanghai, China). DFT calculations of FMOs were carried out by means of the Gaussian 16 Quantum-Chemical calculation software package (Gussian, Inc. Cambridge, UK) with the B3LYP hybridizing functional and the 6–31G+(d,p) basis set.

### 2.4. Photochromic Experiments

Photochromic properties experiments were performed with a 300 W xenon light source (PLS-SXE, Beijing Perfectlight Technology Co. Ltd., Beijing, China) filtered by a UV filter (pass above 400 nm wavelength) as the optical-light source underneath the circumstance of entire light shield. The straight-line distance between the light and PMoA/PANI hybridizing thin film was 150 mm. The PMoA/PANI hybrid thin film samples were exposed to air during the process of the xenon light source illumination. The radiation was increased over time to generate a series curve of the results. Another sample of PMoA/PANI hybridizing thin film was exposed to air but not exposed to light, and absorption spectrogram curve were concerned regularly to supervise the bleaching process. All experiments were performed at room temperature.

## 3. Results and Discussion

**Figure 2:**
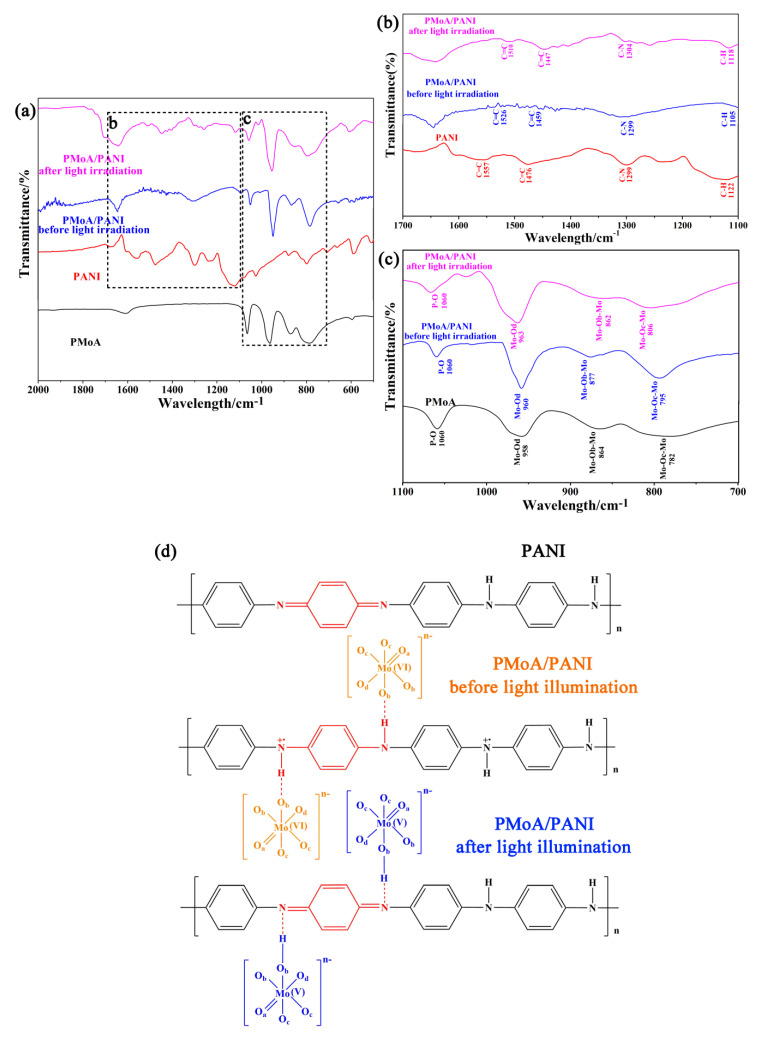
(**a**) FTIR spectrogram of PMoA, PANI, and the PMoA/PANI hybridizing thin film before and after optical-light illumination. (**b**) FTIR spectrogram in the PANI region of PANI and PMoA/PANI before and after optical-light illumination. (**c**) FTIR spectrogram in the PMoA region of PMoA and the PMoA/PANI hybridizing thin film before and after optical-light illumination. (**d**) Chemical structures of PANI and the PMoA/PANI hybridizing thin film before and after optical-light illumination.

The FTIR spectra of the PMoA, PANI, and the PMoA/PANI hybrid thin film before and after optical-light illumination in the wavenumber range of 500 cm^−1^–2000 cm^−1^ are shown in Figure 2a. An enlargement of the FTIR spectrogram in the PMoA region is displayed in Figure 2b. The infrared-active vibrations of ν(Mo-Od), ν(P-O), ν(Mo-Oc-Mo), and ν(Mo-Ob-Mo) were observed in the spectrogram of PMoA and the PMoA/PANI hybrid thin film, and the infrared vibration at 1060 cm^−1^ attributed to ν(P-O) did not change in the spectrum of PMoA and the PMoA/PANI hybrid thin film before and after optical-light illumination, which demonstrated that the PMoA Keggin structure was well maintained in the PMoA/PANI hybrid thin film. 

The vibrations at 795 cm^−1^ attributed to ν(Mo-Oc-Mo) and at 877 cm^−1^ attributed to ν(Mo-Ob-Mo) in the spectrum of PMoA are attributed to the balance vibration, the intensity of which can be used to analyze the interaction between heteropolyacids and polymers. Compared with the peak positions in the spectrum of pristine PMoA, the stretching vibrations of ν(Mo-Oc-Mo) and ν(Mo-Ob-Mo) peaks were blueshifted by 13 cm^−1^ and the ν(Mo-Od) peak redshifted by 2 cm^−1^ in the spectrum of the PMoA/PANI hybridizing thin film. The FTIR spectrogram characterization of the four samples under different conditions indicated that PMoA maintained the base Keggin structure and interacted interfacially with PANI in the PMoA/PANI thin film during the process of protonation. Because Mo-Ob-Mo and Mo-Od served as of the pathways for charge transfer, the electron density was enhanced in the regions of Mo-Ob-Mo and Mo-Oc-Mo, which led to blueshifts of the stretching vibrations. Mo-Od maintains the stability of PMoA. Because of the protonation effect, PMoA lost protons, and the electron density decreased during the interaction with PANI, which caused a redshift in the spectrum, thus verifying the existence of an interfacial interaction between PMoA and PANI, as shown in the chemical structure of the PMoA/PANI hybridizing thin film before light illumination in Figure 2c. Compared with the spectrum of PMoA/PANI before optical-light illumination, the spectrum of the PMoA/PANI hybridizing thin film after optical-light illumination exhibited a decrease in the peaks at 806 cm^−1^ attributed to ν(Mo-Oc-Mo) and at 862 cm^−1^ attributed to ν(Mo-Ob-Mo), which was attributed to the reduction of heteropoly acid to heteropoly blue and proton (hydrogen ion) transfer to PMoA.

An enlargement of the FTIR spectrum in the PANI region is displayed in Figure 2c. The infrared peaks at 1476 cm^−1^ attributed to ν(C=C) and at 1122 cm^−1^ attributed to ν(C–H) are attributed to the PANI benzene structure in pure PANI [24,25], the infrared peak at 1557 cm^−1^ attributed to ν(C=C) is attributed to the PANI quinone structure in pure PANI [26,27,28] and the infrared peak at 1299 cm^−1^ attributed to ν(C–N) is attributed to the PANI benzene-quinone-benzene structure in pure PANI, which reflects the protonation doping position and protonation ratio of PANI [29]. Compared with the spectrum of PANI, the spectrum of the PMoA/PANI hybridizing thin film before optical-light illumination exhibited substantial decreases in the infrared peaks and redshifts of 17 cm^−1^ at 1459 cm^−1^ attributed to ν(C=C) and at 1105 cm^−1^ attributed to ν(C–H), redshifts of 21 cm^−1^ of the infrared peaks at 1526 cm^−1^ attributed to ν(C=C), respectively, and the infrared peak at 1299 cm^−1^ attributed to ν(C–N). These results demonstrated that the basic geometrical structure of the benzene ring for PANI was not destroyed, whereas the quinone-type structure of PANI transformed into a benzene-type structure after protonation by PMoA, and the protonation position was the nitrogen atom in the quinone ring. Correspondingly, compared with the spectrum of the PMoA/PANI hybridizing thin film before optical-light illumination, the spectrum of the PMoA/PANI after optical-light illumination exhibited similar peaks at 1188 cm^−1^, 1447 cm^−1^ and 1510 cm^−1^ attributed to ν(C–H), ν(C=C) and ν(C=C) and a change in the peak at 1304 cm^−1^ attributed to ν(C–N). These results verify that protons (hydrogen ions) transfer to PMoA and that the benzene-type structure converts back to a quinone-type structure after optical-light illumination of the PMoA/PANI hybridizing thin film [24,25,26,27,28], which makes possible the protonation effect generated again in PANI and PMoA, as shown in the chemical structure of the PMoA/PANI hybridizing thin film after light illumination in Figure 2d. 

**Figure 3:**
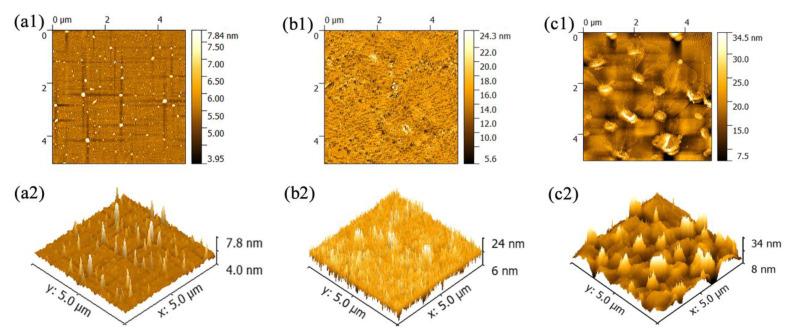
(**a1**,**a2**) AFM 2D and 3D image of PANI, (**b1**,**b2**) AFM 2D and 3D image of the PMoA/PANI hybridizing thin film before optical-light illumination, (**c1**,**c2**) AFM 2D and 3D image of the PMoA/PANI hybridizing thin film after optical-light illumination.

The AFM characterization, which was used to examine the change in surface morphology, is shown in Figure 3. The 2D images of PANI and the PMoA/PANI hybrid thin film before and after optical-light illumination are displayed in Figure 3a1,b1,c1 and show that all three types of thin films are distributed evenly on the treated substrate in the range of 5.0 × 5.0 μm^2^, resulting in the three images having an even distribution color. The 3D images of PANI and the PMoA/PANI hybrid thin film before and after optical-light illumination in the range of 5.0 × 5.0 μm^2^ are exhibited in Figure 3a2,b2,c2. The peaks of the three types of thin films all had average surface sizes, which also confirmed that they are distributed evenly on the treated substrate. The root mean square (RMS) roughness of the PMoA/PANI hybridizing thin film before optical-light illumination was 2.34 nm (compared with 0.305 nm for PANI), which confirmed that the protonation effect of PMoA increased the spatial angle of the PANI polymer. Upon optical-light illumination, the root mean square roughness of the PMoA/PANI hybridizing thin film increased from 2.34 to 3.43 nm, which verified that the formation of heteropoly blue (PMo(V)A) and proton transfer increased the spatial angle of the PANI polymer. The change in morphology in PANI and the PMoA/PANI hybridizing thin film before and after optical-light illumination indicated that the addition of PMoA, protonation and proton transfer during photochromism resulted in an increasingly rough morphology, as shown by AFM. Therefore, the AFM characterization demonstrated that PMoA molecules interact with the PANI polymer via ionic bonding upon protonation of PMoA after the PMoA molecules complex with the PANI polymer, which also changes the spatial arrangement of PANI to narrow the peaks in the PMoA/PANI hybridizing thin film before optical-light illumination.

**Figure 4:**
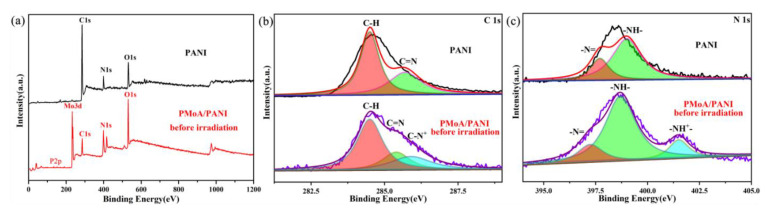
(**a**) XPS photoelectron spectrograms of PANI and PMoA/PANI hybrid thin films before illumination. (**b**) XPS photoelectron spectrogram and Gaussian deconvolution curve fitting for C 1s. (**c**) XPS photoelectron spectrogram and Gaussian deconvolution curve fitting for N 1s.

XPS photoelectron spectrograms of pure PANI and the PMoA/PANI hybrid thin film before illumination are shown in Figure 4a. In the full spectrogram (Figure 4a), all the peaks could be attributed to C, N and O (three different elements) in PANI and P, Mo, C, N and O (five different elements) in the PMoA/PANI hybridizing thin film before illumination; no other elements were observed. The XPS survey scan of C and N significantly reflected the structural change of PANI upon protonation. The C 1s XPS survey spectrogram of PANI and the PMoA/PANI hybrid thin film are displayed in Figure 4b. For pure PANI, the peaks fit the binding energies of C 1s at 284.5 eV, attributed to the C–H of the benzene ring and quinone ring, and 285.7 eV, attributed to the C=N of the quinone ring. For the PMoA/PANI hybridizing thin film, the peaks fit the binding energies of C 1s at 284.5 and 285.4 eV, attributed to the C–H and C=N groups, and a binding energy of C 1s at 285.9 eV, attributed to C–N^+^, was observed upon protonation by PMoA. Furthermore, protonation by PMoA contributed to the redshift of the binding energy of the C=N groups. The N 1s XPS survey spectrogram of PANI and the PMoA/PANI hybridizing thin film are shown in Figure 4c; the peak fit of the binding energies of N 1s at 397.7 and 399.0 eV were attributed to the –N= and –NH– groups of pure PANI, and the bonding energies of N 1s at 397.25 and 398.70 eV were attributed to the –N= and –NH– groups of PMoA/PANI. A new peak observed at 401.5 eV was attributed to the –NH^+^– group, which was caused by protonation by PMoA. Similarly, protonation by PMoA also contributed to the redshift of the binding energy of the –N= and –NH– groups in the spectrogram of the PMoA/PANI hybrid thin film. Thus, protonation by PMoA caused the binding energy of the C–H and C=N groups and the –N= and –NH– groups to redshift. Upon protonation by PMoA, the group consisting of 43.26% C–H and 8.39% C=N was converted to C–N^+^, and the group consisting of 4.27% –N= and 12.77% –NH– was converted to –NH^+^– in PANI of the PMoA/PANI hybridizing thin film. Table 1 shows that the percentages of C–H and C=N groups were 70.80 and 29.20% in pure PANI, and for the PMoA/PANI hybrid thin film, the C–H, C=N, and C–N^+^ groups contributed 55.36, 22.75 and 23.89%, demonstrating that one part of C–H transferred from the quinone ring to the benzene ring and that a portion of the C=N groups were converted to C–N^+^. Similarly, the –N= and –NH– groups contributed 27.89 and 72.11% in pure PANI, and for the PMoA/PANI hybrid thin film, the –N=, –NH–, and –NH^+^– groups contributed 18.21, 64.75 and 17.04%, confirming that a portion of the –N= and –NH– groups are converted to –NH^+^– upon protonation and unipolar rearrangement. The change in the contributions of the functional groups indicated that the site of protonation by PMoA was on the nitrogen atoms of the quinone structure of PANI and spread through the PANI polymer chain and rearranged via the unipolar effect.

**Figure 5:**
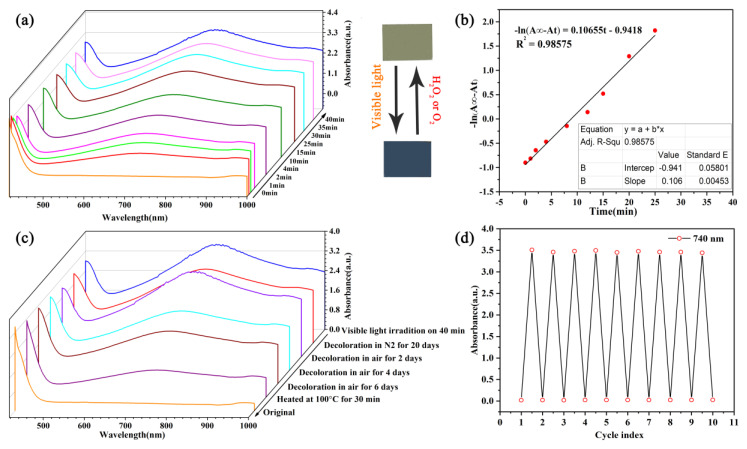
(**a**) UV-Vis spectrogram of the coloration process of the PMoA/PANI hybridizing thin film for different coloration time. (**b**) Kinetic plot of the first-order photochromic process in the PMoA/PANI hybridizing thin film. (**c**) UV-Vis spectrogram of the decoloration process of the PMoA/PANI hybridizing thin film for different decoloration approach. (**d**) The reversibility of the coloration cycle of the PMoA/PANI hybridizing thin film in H_2_O_2_.

The UV-Vis absorption spectrogram of the coloration process of the PMoA/PANI hybrid thin film at wavelengths of 400 nm to 1000 nm for different times are displayed in Figure 5a. Before optical-light illumination, a characteristic absorption by the PMoA/PANI hybridizing thin film in the ultraviolet-optical (UV-Vis) region is observed. Then, after optical-light illumination, the hybrid thin film gradually converted from pale yellow to a deep blue color and the characteristic absorption peak at 732–740 nm was attributed to the intervalence charge transfer (IVCT) [30] transition from Mo^6+^ to Mo^5+^, which was the characteristic absorption in the spectrogram of PMoA blue. Moreover, as the absorption intensity increased, the characteristic peak site of IVCT underwent a redshift from 732 nm to 740 nm with incremental illumination time. Every 10 min test showed that the coloration process was gradual and was not accomplished in one step. The characteristic absorption peak eventually reached coloration saturation with a maximum absorption intensity of 3.46 in 40 min under optical-light illumination, which verified that the illumination time demanded for the absorbance of the PMoA/PANI hybridizing thin film to reach coloration saturation was long and the coloration rate was low, reflecting that the volume of d-d charge transfer was large.

The kinetics of the photochromic coloration process of the PMoA/PANI hybridizing thin film at a wavelength of 740 nm are displayed in Figure 5b. The coloration process can be described by the following first-order kinetic formula:

−*ln*(*A∞* − *At*) = *kt* + b (1)

where *A∞* is the absorbance value when the coloration process is complete, *At* is the absorbance value at each test time during the coloration process, *k* is the coloration rate constant and b is a constant for the formula. The PMoA/PANI hybrid thin film fits the first-order kinetic formula, and the constant *k* is 0.08 min^−1^, which reflects the coloration rate.

The different decoloration processes of the PMoA/PANI hybridizing thin film are exhibited in Figure 5c. The image shows that without any light illumination, the decoloration process proceeds successfully when the PMoA/PANI hybrid thin film is placed in air, which contains a large amount of oxygen, confirming that oxygen performs a key role in the decoloration process. However, when the hybrid thin film is placed in a nitrogen gas environment, the decoloration process does not proceed. When the PMoA/PANI hybridizing thin film was heated at 100 °C for 30 min, the intensity of the absorbance peak decreased to 1.097, and the bleaching process reached 80%.

The invertibility of coloration and decoloration cycle of PMoA/PANI hybridizing thin film at 740 nm is displayed in Figure 5d. The PMoA/PANI hybridizing thin film was irradiated with optical-light during the coloration process, and then 15% H_2_O_2_ was dropped onto the substrate during the decoloration process. The primary absorbance of every cycle increased as the times of illumination cycles increased, attributing to the intensity of the emission of optical-light. Different absorbance between coloration and decoloration was in the range of 3.59 to 3.42 over 10 cycles, indicating that the PMoA/PANI hybrid thin film presented favorable photochromic properties, with high stability and good invertibility [31].

As Table 2 shows, the PMoA/PANI hybrid thin film exhibited a high maximum absorbance compared with the other representative samples, which indicated that the protonation effect of PMoA toward PANI in the PMoA/PANI hybrid thin film could increase the maximum absorbance and greatly improve the photochromic properties of the PMoA/PANI hybrid thin film.

**Figure 6:**
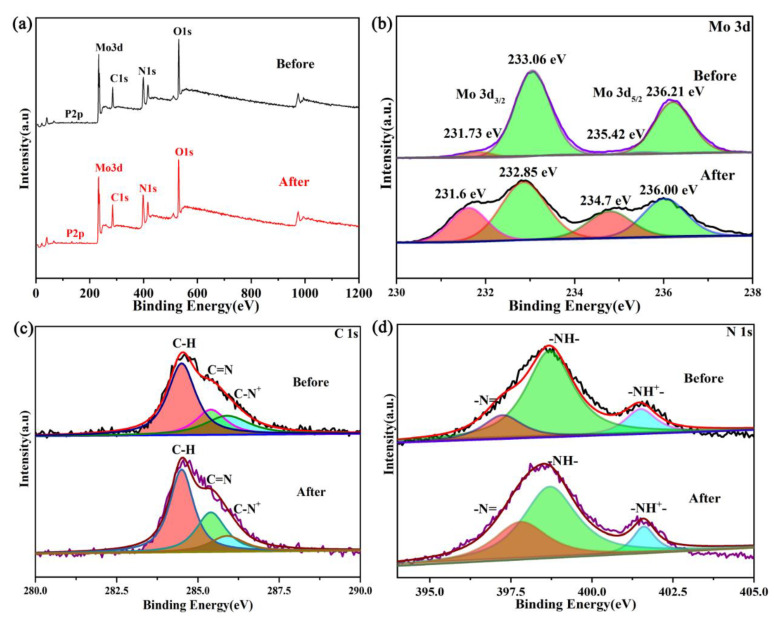
(**a**) XPS survey spectrogram of the PMoA/PANI hybrid thin film before and after optical-light illumination. (**b**) XPS survey spectrogram and Gaussian deconvolution curve fitting for Mo 3d in the PMoA/PANI hybrid thin film. (**c**) XPS survey spectrogram and Gaussian deconvolution curve fitting for C 1s in the PMoA/PANI hybrid thin film. (**d**) XPS survey spectrogram and Gaussian deconvolution curve fitting for N 1s in the PMoA/PANI hybrid thin film.

The XPS spectrogram of the PMoA/PANI hybrid thin film before and after optical-light illumination are displayed in Figure 6a. All the peaks were attributed to P, Mo, C, N and O, and no other elements were observed. Mo XPS spectrogram of the PMoA/PANI hybridizing thin film are shown in Figure 6b. The fitting peaks of the Mo spin-split energy levels correspond to Mo 3d_3/2_ and Mo 3d_5/2_. The binding energies of the PMoA/PANI hybrid thin film before optical-light illumination at 233.06 and 236.21 eV are attributed to Mo^6+^, while the binding energies at 235.58 and 231.73 eV are attributed to Mo^5+^. Similarly, the binding energies of the PMoA/PANI hybrid thin film after optical-light illumination at 232.85 and 236.00 eV are attributed to Mo^6+^, while the binding energies at 234.7 and 231.6 eV are attributed to Mo^5+^. To further investigate the change in electronic structure during the coloration process of PMoA/PANI, Gaussian deconvolution peak integration was used to calculate the respective proportions of Mo^6+^ and Mo^5+^ in the PMoA/PANI hybridizing thin film before and after optical-light illumination.

The relative proportions of Mo^6+^ and Mo^5+^ before and after optical-light illumination are shown in Table 3. The ligand-to-metal charge transfer (LMCT) triggered a change in the valence of Mo, causing Mo^6+^ to be reduced to Mo^5+^. Table 2 shows that the proportion of Mo^5+^ in the PMoA/PANI hybrid thin film was 7%, which was attributed to the X-ray illumination of the PMoA/PANI hybrid thin film before optical-light illumination. The proportion of Mo^5+^ in the PMoA/PANI hybrid thin film after optical-light illumination was 36%, almost 5 times larger than that before optical-light illumination. Compared with that of the PMoA/PANI hybrid thin film before optical-light illumination, the Gaussian deconvolution peak integration area for Mo^5+^ of the PMoA/PANI hybrid thin film after optical-light illumination is obviously increased, which confirmed that under the stimulus of photons, electronic transitions from the original energy level occurred on a large scale, such that a large amount of Mo^6+^ gained electrons to be reduced to Mo^5+^.

The C 1s XPS spectrogram of the PMoA/PANI hybrid thin film before and after optical-light illumination is exhibited in Figure 6c. Compared with the spectrum of the PMoA/PANI hybrid thin film before illumination, that of the PMoA/PANI hybrid thin film after illumination exhibited binding energies at 284.5, 285.4, and 285.9 eV that were attributed to C–H, C–N^+^, and C=N. The relative proportions of C–H, C–N^+^, and C=N change during the photochromic process because of proton transfer, which results in a valence change of the Mo in PMoA. Compared with the relative proportions in the PMoA/PANI hybrid thin film before illumination, the relative proportion of C–N^+^ decreased, that of C=N increased, and that of C–H remained constant after illumination, which surely indicated that the PANI in the PMoA/PANI hybrid thin film was converted from the benzene-type structure of single-polarized PANI back to the quinone-type structure of eigenstate PANI because of proton transfer. The N 1s XPS spectrogram of the PMoA/PANI hybrid thin film before and after optical-light illumination is displayed in Figure 6d. Compared with the spectrogram of the PMoA/PANI hybrid thin film before illumination, that of the PMoA/PANI hybrid thin film after illumination exhibited binding energies at 397.76, 398.70, and 401.5 eV that were attributed to –N=, –NH–, and –NH^+^–. The change in the proportions of –N=, –NH–, and –NH^+^– during the photochromic process was attributed to proton transfer; the relative proportions of –NH– and –NH^+^– decreased, whereas that of –N= increased, which also triggered conversion of the PANI in the PMoA/PANI hybrid thin film from the benzene-type structure of single-polarized PANI back to the quinone-type structure of eigenstate PANI upon proton transfer [44,45].

The change in the valence state of Mo also triggered the transformation of PANI. As Table 4 shows, the proportions of the C–H, C=N and C–N^+^ groups were 53.36, 22.75 and 23.89%, respectively, in the PMoA/PANI hybrid thin film before illumination, and for the PMoA/PANI hybridizing thin film after illumination, the proportions of the C–H, C=N and C–N^+^ groups were 53.36, 32.44 and 14.20%, respectively. A portion of the C–N^+^ groups were reconverted to C=N, but the proportion of C–H did not change in the process. Similarly, the proportions of the –N=, –NH–, and –NH^+^– groups were 18.21, 64.75 and 17.04%, respectively, in PMoA/PANI before illumination, and for the PMoA/PANI hybrid thin film after illumination, the proportions of the –N=, –NH–, and –NH^+^– groups were 31.89%, 58.30% and 9.81%, respectively. Moreover, a portion of the -NH^+^- groups were reconverted to –N= and –NH– upon proton transfer. The change in the proportions of these functional groups indicated that site of proton transfer from PMoA was the nitrogen atoms of the quinone structure of PANI and spread through the PANI polymer chain and rearranged via the unipolar effect.

**Figure 7:**
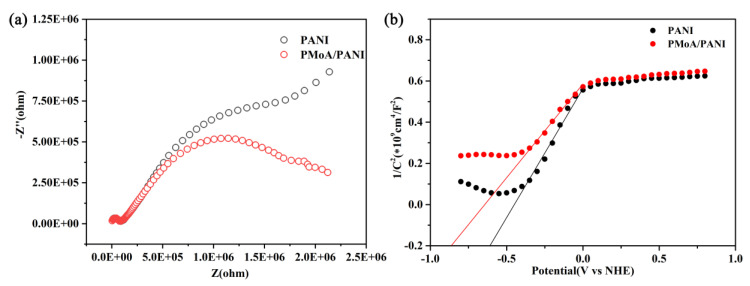
Electrochemistry study of EIS of PANI and the PMoA/PANI hybrid thin film (**a**) EIS of PANI and the PMoA/PANI hybrid thin film. (**b**) Mott-Schottky curve of PANI and the PMoA/PANI hybrid thin film.

The EIS results of PANI and the PMoA/PANI hybrid thin film are shown in Figure 7a. The electrochemical impedance arc radius of PANI was larger than that of the PMoA/PANI hybrid thin film, which indicated that electrons delocalized more easily in the PMoA/PANI hybrid thin film than in PANI after protonation, providing strong evidence that protonation enhanced the electron transfer efficiency in the PMoA/PANI hybrid thin film.

For further investigating the electron transfer efficiency, the Mott-Schottky curve of PANI and the PMoA/PANI hybrid thin film are given in Figure 7b. After estimation by the Mott-Schottky formula, the flat-band potential of PANI was −0.61 V, and flat-band potential of PMoA was −0.86 V. The flat-band potential of PMoA/PANI was lower than that of PMoA, indicating the PMoA/PANI had more tendency to be donate electrons than PANI and protonation increased the electron transfer properties in the PMoA/PANI hybrid thin film.

**Figure 8:**
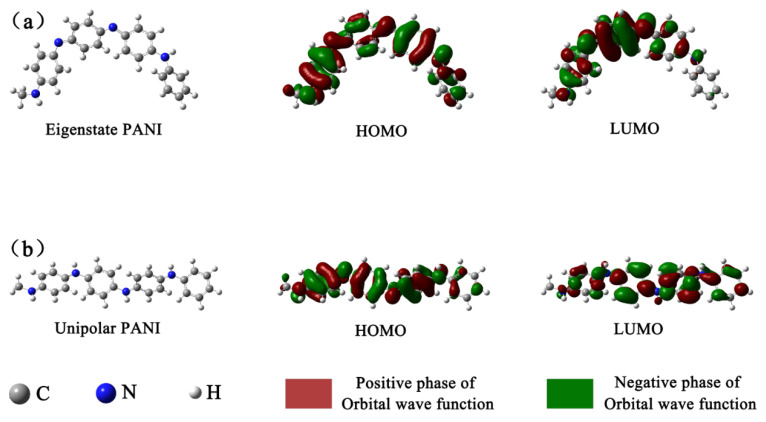
(**a**) DFT calculations of the FMOs of the eigenstate PANI unit. (**b**) DFT calculations of the FMOs of the unipolar PANI unit.

The FMOs of eigenstate PANI and unipolar PANI calculated by DFT are displayed in Figure 8. Figure 8a shows that in the eigenstate PANI unit, the electron density in the highest occupied molecular orbital (HOMO) was concentrated on benzene rings 1 and 3 and the nitrogen atoms connected to them. Conversely, the electron density in the lowest unoccupied molecular orbital (LUMO) was concentrated on quinone ring 2 and the nitrogen atoms connected to it. Upon electron transfer from the HOMO to the LUMO, the electron density on quinone was increased in the eigenstate PANI unit. Figure 8b shows that in the unipolar PANI unit arising from protonation of eigenstate PANI, the electron density of the HOMO was distributed evenly across benzene rings 1, 2 and 3 and the nitrogen atoms connected to them. Furthermore, the electron density of the LUMO was distributed evenly across benzene rings 1 to 4 and the nitrogen atoms connected with them. Upon electron transfer from the HOMO to the LUMO, the electron density on the quinone ring was increased in the unipolar PANI unit. In the FMOs of eigenstate PANI and unipolar PANI, all nitrogen atoms exhibit obvious π properties, and those hybridizing atoms evidently contributed to electron delocalization across the four rings of the polymer. Moreover, compared with eigenstate PANI, unipolar PANI has a more homogeneous electron density distribution regardless of whether a benzene ring or a nitrogen-containing ring is present. The HOMO consisting of orbitals on the ring structure exhibits π properties, facilitating electron delocalization. In addition, the LUMO consisting of orbitals on the ring structure exhibits π* properties. The FMO calculation provides strong evidence that the unipolar PANI possesses strong electron delocalization and transfer abilities via protonation of eigenstate PANI, which was demonstrated by the EIS test and confirmed the hypothesis that the PANI/PMoA hybridizing thin film exhibits improved photochromic properties [46].

**Figure 9:**
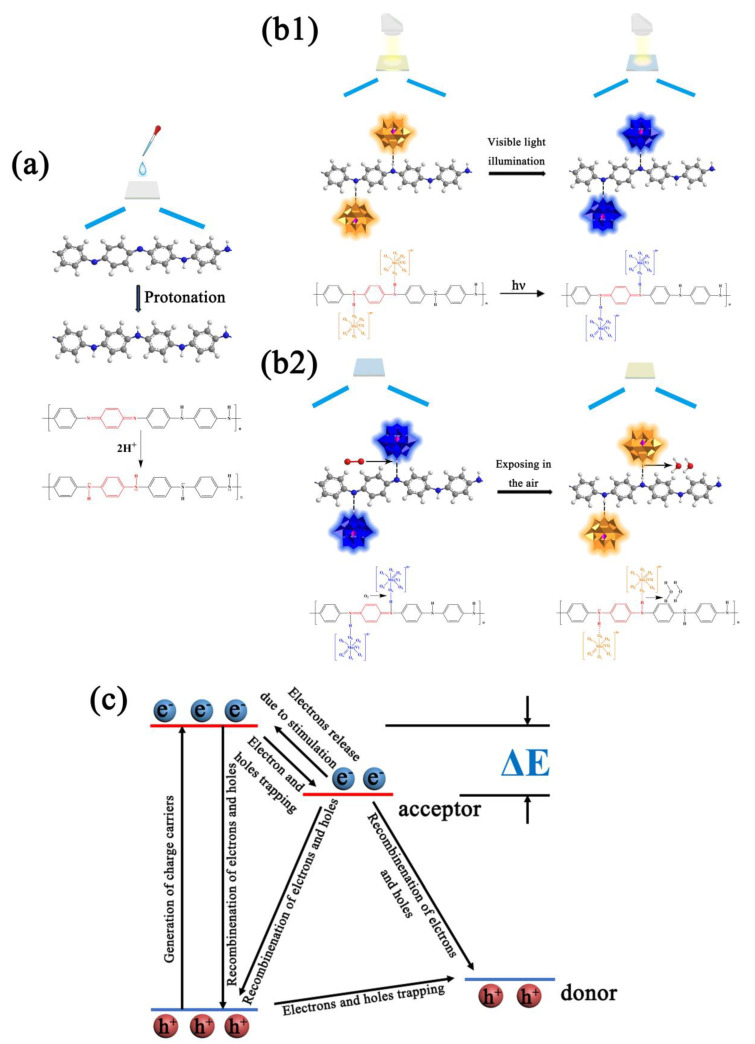
(**a**) The protonation process of PANI. (**b1**) Process of coloration of the PMoA/PANI hybrid thin film. (**b2**) Process of decoloration of the PMoA/PANI hybrid thin film in air. (**c**) Schematic diagram of LMCT.

According to the mechanism of photochromism for PMoA and the application of PANI, the mechanism photochromism of the PMoA/PANI hybrid thin film was attributed to IVCT and LMCT [30] of PMoA and the change in electrical conductivity of PANI via protonation. As shown in Figure 9a, the eigenstate PANI consisted of benzene and quinone with a ratio of approximately 3:1 in every polymer unit, resulting in poor electrical conductivity that was approximately equal to that of an insulator before protonation [47]. When PANI was combined with PMoA, the chemical structure of PANI underwent a series of transformations due to protonation by PMoA. Furthermore, the electrons associated with nitrogen atoms in amino groups (–N=) delocalized to the adjacent benzene ring, causing the density of the electron cloud to decrease; such electron delocalization induced a conjugation effect, which caused the electron cloud on the PANI chain to rearrange, as shown in the FTIR results in Figure 2. 

This rearrangement of the π electrons triggered a transformation of the quinone-bipolaron state into the benzene monopolaron state. The benzene monopolaron structure exhibited a semi-full conductive band, which resulted in electrons moving easily within the energy band when stimulated, enhancing the electrical conductivity of PANI. Moreover, PMoA interacted with PANI via ionic bonds, as shown in Figure 9b1.

As Figure 9c shows, since PMoA has a special Keggin-type structure [48], the Mo^6+^ ion in oxidized PMoA has a d^0^ electronic structure, which causes PMoA to exhibit a single absorption peak in the ultraviolet and visible region that is attributed to the IVCT [30] effect, and then low-energy electrons mainly exist in the 2p orbital of the O atoms of PMoA. Upon visible light irradiation of the PMoA/PANI hybrid film, the electrons in the LUMO, consisting of the 2p orbital of the O atoms, were excited to the HOMO and then captured by electron trapping of the middle energy band formed by metal ions, converting d^0^ electrons to d^1^ electrons [49,50] by LMCT. Then, the d^1^ electron were transferred through the molecule because of different valence states and d-d charges of adjacent metal centers such that Mo^6+^ ions were reduced to Mo^5+^ ions by IVCT, which promoted the absorption of visible light [30]. Moreover, the bridged oxygen (Od) of PMoA has a unique redox absorption point, and protons (H^+^) can easily be absorbed by Ob to compensate for the negative charge, resulting in a LUMO with antibonding properties [51]. The terminal oxygen (Od) of the Keggin-type structure, which is linked to Mo by double bonds, also possessed the strongest chemical activity in PMoA.

As Figure 9b1,b2 show, in general, combining the unique electron transfer ability of PMoA and the proton transfer ability of PANI, the photochromic mechanism of the PMoA/PANI hybrid film can be described as follows: PANI is protonated by multiproton PMoA in the liquid phase; then, the eigenstate of the quinone-type structure in every polymer unit of PANI is converted to a single-polarized benzene-type structure, the electrons in the single-polarized benzene structure are easily delocalized, N-H^+^ groups establish in every protonated PANI polymer unit, and PMoA interacts firmly with PANI by ionic bonds. Upon optical-light illumination of the PMoA/PANI hybrid film, the PMoA undergoes IVCT and LMCT [30] to reduce Mo^6+^ to Mo^5+^, and the color of the PMoA/PANI hybrid film changes from pale yellow (PMoA(Mo^6+^)) to blue (PMoA(Mo^5+^)). Furthermore, because of the LMCT effect, the electrons in N-H^+^ are transferred to the LUMO, consisting of the 2p orbitals of O atoms, and H^+^ are captured by Ob, protons and transferred such that PANI loses protons. Thus, the benzene-type structure is reduced back to the quinone structure, while PMoA comes into contact with PANI via hydrogen bonding. Because of the strong electron delocalization and transfer properties of unipolar PANI, the d^1^ electrons are transferred through the molecule due to the different valence states and d-d charges of adjacent metal centers, which vastly enhances the maximum absorbance of optical-light, significantly improving photochromic properties of the PMoA/PANI hybrid film. The electrons must overcome ionic bonding to be transferred to the LUMO, consisting of O 2p orbitals, and the coloration process becomes slower. During the decoloration process, the PMoA/PANI hybrid films are exposed to air, and PMoA(Mo^5+^) reacts with O_2_ and is then reoxidized to PMoA(Mo^6+^). PMoA(Mo^6+^) interacts with PANI again, resulting in a cycle of coloration and decoloration of PMoA(Mo^5+^)/PMoA(Mo^6+^) in the PMoA/PANI hybrid film.

## 4. Conclusions

A photochromic organic/inorganic PMoA/PANI hybrid thin film was easily synthesized by the protonation effect and demonstrated great photochromic properties, especially high light absorption of 3.46 in the UV-vis spectrum. AFM presented an even distribution of PMoA/PANI hybrid thin film root mean square roughness with 2.34 nm before illumination and 3.43 nm after illumination. All the characterizations and mechanistic analyses showed that protonation played a key role in the transformations of the quinone structure of eigenstate PANI and the single-polarized benzene structure of PANI observed by FTIR and XPS, which induced absorption by PMoA, enhancing the electrons transfer property verified by electrochemistry and DFT studies and providing electrons for PMoA to alter the valence state in order to achieve exceptional photochromic performance. All these results provided a new possibility for the application of PMoA and PANI, especially in the region of laser ink-free printing and optical control switches.

## Figures and Tables

**Table 1:** Comparison of C– and N-related functional groups of pure PANI and the. PMoA/PANI hybrid film before irradiation.

**Sample**	**Pure PANI**					
Tested element	C			N		
Functional group	C–H	C=N	C–N^+^	–N=	–NH–	–NH^+^–
Binding Energy (eV)	284.5	285.7	-	397.7	399.0	-
Contribution	70.80%	29.20%	-	27.89%	72.11%	-
**Sample**	**PMoA/PANI Hybrid Film before Irradiation**					
Tested element	C			N		
Functional group	C–H	C=N	C–N^+^	–N=	–NH–	–NH^+^–
Binding Energy (eV)	284.5	285.4	285.9	397.25	398.70	401.5
Contribution	53.36%	22.75%	23.89%	18.21%	64.75%	17.04%

**Table 2:** Comparison of the maximum absorbance of photochromic materials.

Photochromic Material	Maximum Light Absorbance	Reference
Fe(III) R-PLG_23_ metal complex	0.12	[32]
PMoA/TiO_2_	0.13	[31]
PMoA/Na-MMT/PVPd	0.19	[33]
PMoA/PVPd	0.225	[14]
CP/TiO_2_	0.25	[34]
(NH_4_)_14_[NaP_5_W_30_O_110_]	0.28	[35]
Gold Nanoparticle-Molybdenum Trioxide Thin Films	0.75	[36]
CsPbBr_3_ Quantum Dot Films	0.78	[37]
MoO_3_ Nanoribbons	0.8	[9]
Two Tri-Lacunary α-Dawson-Type Polyoxotungstates	1.25	[38]
Non-Stoichiometric Monoclinic Structured Tungsten Trioxide (WO_3−*x*_)	1.27	[39]
Spiropyran-Containing Fluorinated Polyacrylate Hydrophobic Coatings	1.5	[40]
PEG-400 assisted WO_3_–TiO_2_–ZnO films	1.6	[41]
WO_3−x_ QDs	2.8	[34]
Rhodamine Joined with Polyurethane	3.2	[42]
Aromatic Sulfonium Octamolybdates	3.35	[43]
PMoA/PANI Hybridizing Thin Film	3.46	**This work**

**Table 3:** The change in the valence state of Mo in the PMoA/PANI hybrid thin film.

Sample	Mo^5+^		Mo^6+^		Mo^5+^/Mo Ratios
	3d_3/2_	3d_5/2_	3d_3/2_	3d_5/2_	
Before	231.85	235.50	233.05	236.20	0.07
After	231.65	234.80	232.90	236.0	0.36

**Table 4:** Comparison of C– and N–-containing functional groups of PMoA/PANI hybrid thin films before and after optical-light illumination.

**Sample**	**PMoA/PANI Hybrid Thin Film before Illumination**					
Tested element	C			N		
Functional group	C–H	C=N	C–N^+^	–N=	–NH–	–NH^+^–
Binding Energy (eV)	284.5	285.4	285.9	397.2	398.2	401.5
Contribution	53.36%	22.75%	23.89%	18.21%	64.75%	17.04%
**Sample**	**PMoA/PANI Hybrid Thin Film after Illumination**					
Tested element	C			N		
Functional group	C–H	C=N	C–N^+^	–N=	–NH–	–NH^+^–
Binding Energy (eV)	284.5	285.4	285.9	397.80	398.70	401.5
Contribution	53.36%	32.44%	14.20%	31.89%	58.30%	9.81%
